# Effect of the Inter-Tooth Distance and Proximal Axial Wall Height of Prepared Teeth on the Scanning Accuracy of Intraoral Scanners

**DOI:** 10.3390/jfb15050115

**Published:** 2024-04-25

**Authors:** So-Yeun Kim, Keunbada Son, Soo Kyum Bihn, Kyu-Bok Lee

**Affiliations:** 1 Department of Prosthodontics, School of Dentistry, Kyungpook National University, Daegu 41940, Republic of Korea; soyeunkim179@gmail.com (S.-Y.K.); nicejiya527@naver.com (S.K.B.); 2 Advanced Dental Device Development Institute, Kyungpook National University, Daegu 41940, Republic of Korea; oceanson@knu.ac.kr

**Keywords:** intraoral scanner, scanning accuracy, tooth preparation, inter-tooth distance, proximal axial wall height

## Abstract

This study aimed to analyze the effect of the height of the proximal axial wall of the prepared tooth and the distance between the adjacent tooth and the prepared tooth on the scan accuracy of intraoral scanners. Ten working casts with maxillary first molars prepared to receive zirconia crowns were randomly obtained from a dental clinic. Each of the 10 casts was scanned using two intraoral scanners (i700; MEDIT and CS3600; Carestream; computer-aided design [CAD] test model, CTM; N = 15 per working cast) 15 times per scanner. Individual dies of the prepared teeth were fabricated, and high-precision scan data were acquired using a laboratory scanner (CAD reference model, CRM; N = 1). CTMs were aligned relative to the prepared tooth of CRMs by using three-dimensional inspection software (Ver 2018.1.0; Control X; 3D Systems). Data were statistically analyzed using an independent t-test and one-way analysis of variance for between-group comparisons (α = 0.05). The inaccuracy in the proximal regions (mesial or distal) of the prepared tooth was higher than that in the buccal and lingual regions (*p* < 0.05). The scan accuracy was not correlated with the variables when the distance between the adjacent tooth and the prepared tooth was ≥2.0 mm and the height of the proximal axial wall of the prepared tooth was <3.0 mm (*p* > 0.05). Therefore, an excellent scan accuracy can be obtained using an intraoral scanner when the distance between the adjacent tooth and the prepared tooth is ≥2.0 mm and the proximal axial wall height of the prepared tooth is <3.0 mm.

## 1. Introduction

Digital dentistry has achieved revolutionary strides, and with technological advancements, the performance of intraoral scanners (IOSs) has consistently improved [1]. In fixed prosthodontics, intraoral scanning is an alternative to physical impression making [2]. Acquiring a virtual working cast using an IOS is crucial while preparing fixed dental restorations, and scanning accuracy must be evaluated to verify its precision [3]. Scanning accuracy is commonly evaluated by calculating the deviation between a reference virtual model with a guaranteed scan accuracy and a virtual model obtained for testing [4]. Measurements are performed under various experimental conditions in digital dentistry [1,2,3,4]. Under controlled conditions, deviations are primarily attributed to limitations in scanner technology, such as the scanner’s resolution, the software’s capability to stitch complex data points, and the optical properties of the materials used in the scan process [4].

Tooth preparation methods vary depending on the treatment purpose while fabricating fixed dental restorations [5,6]. Because the extent of tooth preparation is dependent on lesion size and restorative material, the gap between the prepared tooth and adjacent teeth can considerably vary [7]. In addition, the amount of tooth prepared in clinical settings differ from standard recommendations based on tooth preparation techniques and operator skills [8]. In a digital workflow, the prepared tooth and adjacent teeth are scanned intraorally to fabricate fixed dental restorations [9,10]. However, the optical source of the IOS cannot easily reach the target because of the high proximal axial wall and the narrow space between the adjacent tooth and the prepared tooth; as a result, scan data are inaccurate [9,10]. Additionally, shadowing effects from high axial walls and tight proximal spaces can obstruct the scanner’s line of sight, leading to data gaps and necessitating manual data manipulation or rescanning, which compromises the workflow efficiency and accuracy [10]. For this reason, the axial height of the prepared tooth should be sufficient for the optimal retention and support of the restoration [11]. Therefore, studies based on the distance from the adjacent tooth and the height of the proximal axial wall should be performed to verify the accuracy of the intraoral scan according to variations in tooth preparations in clinical practice [12]. Furthermore, the research should explore the interplay between the physical properties of the scanners and the geometrical configurations of the prepared teeth, which could provide insights into designing more effective dental restoration strategies.

IOSs have varied mechanical configurations of the optical system and the software-based technology, which is used to align the scanned data and reconstruct the dental arch; thus, various IOSs are used to evaluate scan accuracy [13,14,15,16]. However, the use of IOSs is hampered by varying dental clinical conditions such as wet conditions, limited jaw movement, and narrow and depth of the lesion under the patient’s oral use [17,18,19,20]. The interference caused by these factors necessitates a robust scanner calibration protocol and adaptive scanning strategies to improve data accuracy and reliability [19].

The purpose of this study was to quantify the height of the proximal axial wall and the spacing between the prepared tooth and its adjacent counterparts in patient dental casts, with the goal of determining the impact of these measurements on the accuracy of intraoral digital scans. The following null hypotheses were verified: (1) the scan accuracy of the two IOSs would not be different; (2) the scan accuracy in the proximal (mesial and distal), buccal, and lingual regions of the prepared tooth would not be different, thus indicating the effect of the adjacent teeth on the scanning accuracy of tooth preparations; and (3) the height of the proximal axial wall of the prepared tooth or the distance between the adjacent tooth and the prepared tooth would not affect the scan accuracy.

## 2. Materials and Methods

**Figure 1:**
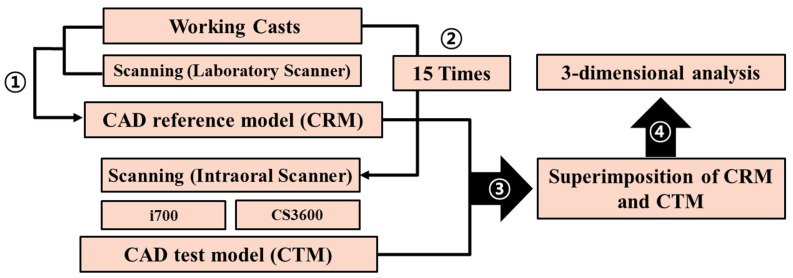
Schematic of the evaluation method used in this study. The numbers indicate the sequence of the experiments.

This research received approval from the Institutional Review Board of Kyungpook National University Dental Hospital for the collection of working casts employed in clinical settings (IRB No. KNUDH-2019-02-02-02, 2 April 2019). It was carried out in compliance with the Helsinki Declaration and the Good Clinical Practice guidelines. Evaluation was conducted as illustrated in Figure 1. Written informed consent for the use of working casts was provided by all patients, and findings were reported in compliance with the applicable Consolidated Standard of Reporting Trials guidelines.

Working casts were obtained from 10 patients. Each patient had a maxillary first molar prepared for a zirconia crown, while the adjacent teeth were retained. The prepared working cast had a chamfer finish line with 1.5 mm reduction and was positioned vertically within 0.5 mm below the gingival. The 10 working casts used in this study were prepared in accordance with the procedure used in a conventional dental laboratory workflow. A laboratory scanner (E1; 3Shape A/S, Copenhagen, Denmark) was utilized. The height of the proximal axial wall of the prepared tooth and the spacing between the adjacent tooth and the prepared tooth were gauged using three-dimensional (3D) inspection software (Ver 2018.1.0; Geomagic Control X; 3D Systems, Rock Hill, SC, USA). The utilization of high-resolution 3D inspection tools allows for precise spatial measurements and a detailed visualization of the topographic features of tooth preparations, which are crucial for ensuring the accuracy of the subsequent scanning process.

**Figure 2:**
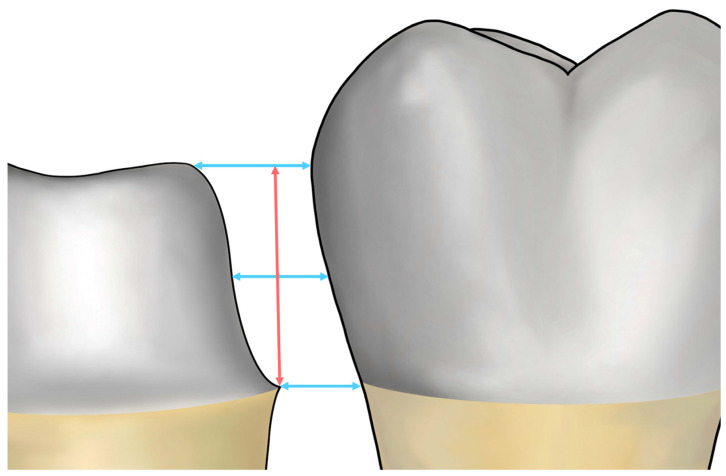
Diagram illustrating the measurement of the distance between the adjacent tooth and the prepared tooth (indicated by blue arrows) and the axial wall height of the prepared tooth (marked by a red arrow) on a virtual plane.

The distance between the adjacent teeth and the prepared tooth was measured in the mesiodistal direction, where the prepared tooth met the adjacent tooth parallel to the occlusal plane. This parameter was determined at three specific points: (1) from the highest point on the occlusal surface of the prepared tooth to the adjacent tooth; (2) at the finish line of the tooth preparation, on a virtual plane passing from the central groove of the adjacent tooth to the prepared tooth; and (3) midway between the first two points (Figure 2). The height of the proximal axial wall of the prepared tooth was set as the average distance between the imaginary lines parallel to the occlusal plane at the highest point and the finish line of the tooth preparation (Figure 2). Based on these measurement criteria (Figure 2), all measurements were completed by a single skilled investigator (K.S.).

Each of the 15 working casts underwent 15 scans on each of the utilized scanners (i700 [MEDIT, Seoul, Republic of Korea] and CS3600 [Carestream Dental, Atlanta, GA, USA] IOSs), resulting in a total of 15 scans per cast with each scanner (computer-aided design [CAD] test model, CTM; N = 15 for each working cast). To mimic a clinical setting, each cast was affixed to the maxilla and mandible of a dental mannequin head (Simple Manikin III, NISSIN, Kyoto, Japan) using temporary adhesives, and the head was then attached to a dental unit chair system (Maxpert; SHINHUNG, Seoul, Republic of Korea). All intraoral scanning was conducted under consistent environmental conditions, including lighting, temperature, and humidity. The occlusal, buccal, and lingual surfaces, with emphasis on the prepared tooth, were scanned first. This was followed by scanning the mesial and distal adjacent teeth. Comprehensive scanning covered all mesial, distal, buccal, and lingual surfaces of the prepared tooth from the occlusal surface down to the finish line. A single experienced operator performed all scans using both IOSs. Ensuring uniform scanning procedures across all samples under controlled environmental conditions minimizes the introduction of variability due to external factors, thus providing a robust dataset for analyzing scanner performance and scan accuracy.

The individual dies of the prepared teeth were fabricated from the working casts to obtain precision scans. The high-precision scan data of individual dies were acquired using a laboratory scanner (E1; 3Shape A/S, Copenhagen, Denmark; CAD reference model, CRM; N = 1) calibrated in accordance with the recommendations of the manufacturer, which guarantees a scan accuracy of <10 µm. The adoption of a high-precision laboratory scanner for reference measurements establishes a reliable baseline for assessing the performance of intraoral scanners and accurately quantifying any deviations.

**Figure 3:**
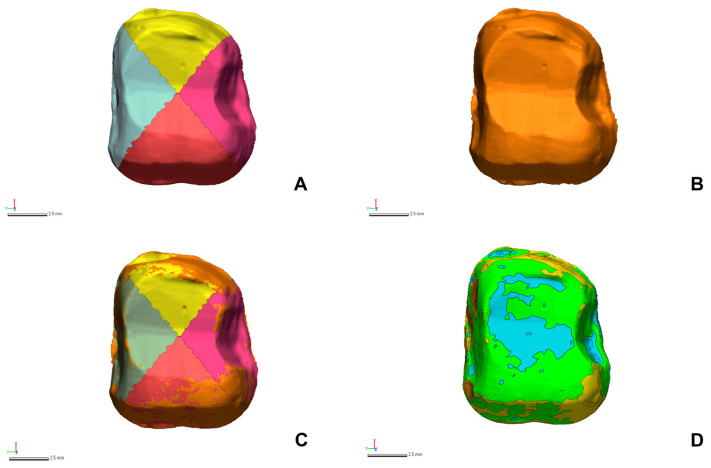
Three-dimensional analysis of the scanning accuracy. (**A**) Segmentation into four regions (mesial, distal, buccal, and lingual regions) in reference data. (**B**) Test data. (**C**) Superposition of the reference and test data. (**D**) The color difference map displays green within a 10 µm deviation and extends to a color range of up to 100 µm.

CTMs (N = 15 per working cast) and CRMs (N = 1 per working cast) were extracted as standard tessellation language (STL) files from each scanner to analyze the scanning accuracy (Figure 3). In the extracted STL files, all areas except the tooth preparation area were deleted, and scanning accuracy was evaluated on the 3D area by using the analysis software (Figure 3). CRMs and CTMs from each working cast group were imported into the software (Figure 3). On the basis of the finish line of CRMs, the tooth preparation area was segmented, and all divided tooth preparation areas were matched with the corresponding areas of the CTMs. CTMs were precisely aligned following a sequence of initial alignment and best-fit alignment, according to the segmented tooth preparation area of CRMs (Figure 3). All point clouds in the 3D area of the segmented tooth preparation of the CRM were evaluated for the mean distance deviation of the point clouds of the corresponding CTMs (Figure 3). The root-mean-squared (RMS) value assessed was computed using the equation below:

(1) $$RMS=\frac{1}{\sqrt{n}}\cdot \sqrt{\sum_{i=1}^{n}{\left(X_{1,i}-X_{2,i}\right)}^{2}},$$

where $X_{1,i}$ represents the coordinate position of the *i*th point in the 3D area of the segmented tooth preparation on the CRM, and $X_{2,i}$ denotes the coordinate position of the *i*th point in the corresponding CTMs. RMS is calculated as the mean of the absolute distance deviations between all point clouds and the corresponding CTMs in the 3D area of the segmented tooth preparation on the CRM. This 3D deviation was depicted as a color difference map (±100 µm color range and ±10 µm in green; Figure 3) to evaluate the deviation. Utilizing RMS as a metric allows for a quantitatively precise measurement of the comprehensive accuracy across the entire scan area, providing a holistic view of the performance of the intraoral scanners.

The divided tooth preparation region of the CRM was segmented into four regions to evaluate the scanning accuracy of the four additional regions (buccal, lingual, mesial, and distal), and RMS values for each region were calculated (Figure 3).

All collected data were confirmed using statistical software (SPSS version 25.0; IBM Corp., Armonk, NY, USA). The distribution of the data was assessed with the Shapiro–Wilk test to verify normalcy. For comparisons between groups, the data underwent statistical analysis through the use of independent t-tests and one-way analysis of variance. Differences among groups were identified using Tukey’s post hoc test (α = 0.05). The effects of the distance between abutments, axial heights, and type of IOS on our outcomes were assessed with multivariate analysis of variance (MANOVA). The correlation between scanning accuracy and the distance to the adjacent teeth or the height of the proximal axial wall of the prepared tooth was analyzed using the Pearson correlation coefficient (α = 0.05). The comprehensive statistical analysis enables the detection of significant differences and trends within the data, providing robust conclusions regarding the factors affecting intraoral scan accuracy.

## 3. Results

Table 1 shows the height of the proximal axial walls of the prepared tooth and the distance between the adjacent tooth and the prepared tooth. The mean distance between the adjacent tooth and the prepared tooth was 1.7 ± 0.4 mm both mesially and distally, which had no significant differences (*p* = 0.532). Moreover, the mesial (3.1 ± 0.8 mm) and distal (3.0 ± 0.9 mm) proximal axial wall heights of the prepared tooth did not significantly differ (*p* = 0.758).

**Figure 4:**
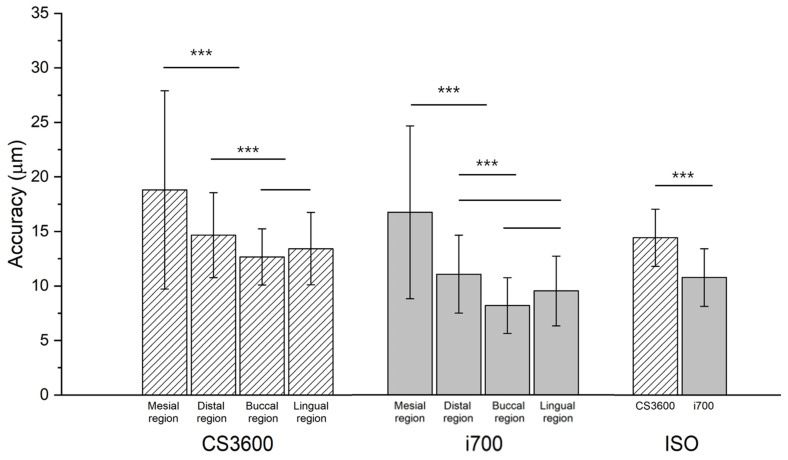
Comparison of the accuracy between the two IOSs in the four regions (mesial, distal, buccal, and lingual regions). *** Significant differences from two groups connected by a line in Tukey’s honest significant difference test, *p* < 0.001. ISO, intraoral scanner.

**Figure 5:**
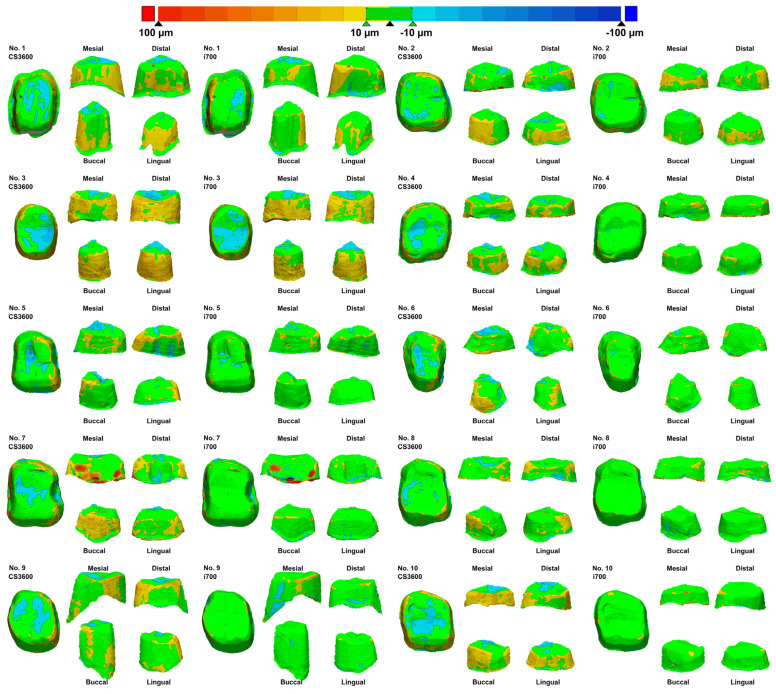
Color difference maps of the 10 working casts acquired using the two intraoral scanners. The color difference map showing green within 10 µm and color range of 100 µm.

The two types of IOSs significantly differed (*p* < 0.001; Figure 4). In all 10 working casts, i700 scans showed an excellent scan accuracy (Table 2). In all working casts and two IOSs, the scanning accuracy significantly varied in the four regions, namely buccal, lingual, mesial, and distal (*p* < 0.05; Figure 4). Specifically, the RMS values in the proximal regions (mesial or distal) were higher than those in the buccal and lingual regions (*p* < 0.05; Table 2). Consistently, the color difference map showed the same results (Figure 5). The color deviation of all 10 working casts in i700 scans was less than that in CS3600 scans (Figure 5).

The assessment of the correlation between scanning accuracy and the distance to the adjacent tooth or the height of the proximal axial wall of the prepared tooth revealed that the scan accuracy was not correlated with the variables when the distance between the adjacent tooth and the prepared tooth was >2.0 mm or the height of the proximal axial wall of the prepared tooth was <3.0 mm (*p* > 0.05; Table 3). However, the scan accuracy showed a significantly negative correlation with the variables (*p* < 0.001; Table 3) with a distance of <2.0 mm between the adjacent tooth and the prepared tooth and a height of >3.0 mm of the proximal axial wall of the prepared tooth.

MANOVA revealed that each factor (distance between abutments, axial heights, and IOS type) significantly affected the accuracy of intraoral scans (*p* < 0.001). These factors also exhibited significant interaction effects on one another (*p* < 0.001).

## 4. Discussion

This study aimed to measure the height of the proximal axial walls of the prepared tooth and the distance from the prepared tooth to the adjacent teeth by using working casts of patients and evaluate how these variables influence the accuracy of intraoral scans. The first null hypothesis, i.e., the scan accuracy of the two IOSs had no differences, was rejected (*p* < 0.05). The scan accuracy of the i700 IOS was superior to that of the CS3600 IOS in all 10 working casts (Table 2). The second null hypothesis, i.e., scan accuracy had no differences in the proximal regions (mesial and distal) and the buccal and lingual regions, was also rejected. The scan data of the proximal regions (mesial and distal) had a significantly increased inaccuracy (*p* < 0.05; Table 2). This finding might show the influence of the adjacent teeth on the scanning accuracy. Furthermore, the third null hypothesis, i.e., the height of the proximal axial wall of the prepared tooth and the distance between the adjacent tooth and the prepared tooth did not affect the accuracy of the scans, was also rejected (*p* < 0.05; Table 3). The results highlighted the significant effect of the distance between abutments, axial heights, and IOS type on the accuracy of intraoral scans. The relationships between these factors and their combined effects on scan accuracy were comprehensively evaluated using MANOVA. The results indicated that each factor independently contributed to the overall scan accuracy, emphasizing the importance of considering these variables in scan result interpretation and appropriate IOS selection for clinical use. Moreover, the study underlines the need for adaptive strategies in intraoral scanning procedures to accommodate anatomical variations and optimize scan outcomes in complex dental topographies. This may include the development of specialized scanning protocols or the use of advanced software algorithms that can better manage data from difficult-to-scan areas.

Tooth preparations of fixed dental prostheses vary according to the treatment purpose for each patient. Furthermore, the extent of preparation primarily depends on the lesion size and the materials used for the prosthesis. For instance, a previous study reported that the depth of preparation in the labial surface for metal–ceramic crowns ranges from 1.28 mm to 1.45 mm depending on the tooth preparation technique [5]. In the present study, the mean distance between the adjacent tooth and the prepared tooth evaluated mesially and distally was 1.7 ± 0.4 mm. In previous clinical studies, the depth of the tooth preparation in the axial wall for zirconia crowns was targeted at 1–1.5 mm [6,7]. In the present study, working casts of teeth prepared for zirconia crowns were used, and the distance from the adjacent tooth, not the amount of tooth reduction, was measured (Table 1). In another study, 392 teeth prepared in the posterior region to receive zirconia crowns were evaluated, and a high rate of problems, such as finish line quality and undercut at the axial wall in the prepared teeth, was obtained [8]. As theoretical tooth preparation differs from the actual clinical situation, the clinical working casts of patients were examined in the present study.

Previous research assessed scanning accuracy by deliberately modifying the spacing between the adjacent tooth and the prepared tooth [9,10]. This study found that the accuracy of scans across proximal (mesial and distal) and buccal and lingual surfaces was not significantly different when the spacing exceeded 3.5 mm [9]. Since the working casts in this study were sourced from actual clinical environments, the greatest observed distance between the adjacent tooth and the prepared tooth was 2.3 mm. The findings suggest that in real-world clinical settings, where the distance between the adjacent tooth and the prepared tooth rarely exceeds 3.5 mm, the proximity of adjacent teeth invariably influences scanning accuracy. Another investigation evaluated scanning precision relative to the distance (0.6, 0.8, and 1.0 mm) from adjacent teeth during tooth preparation for inlays, confirming that proximity impacts accuracy. Thus, the proximity of adjacent teeth potentially affects the scanning accuracy of intraoral scans, regardless of the type of tooth preparation, as evidenced by both the ten different working casts for zirconia crowns in this study and previous studies on inlays.

The axial wall height of the prepared tooth for a fixed dental prosthesis should provide optimal retention and support for the restoration; as such, an appropriate height should be set [11]. A previous study revealed that the axial wall height of the prepared tooth with a total occlusal convergence ranging between 10 and 20 degrees should be ≥4 mm in molars [8]. However, another study pointed out that 77% of 74 first molars prepared to receive zirconia crowns have an axial wall height < 4 mm [8]. In the present study, the mean height of the proximal axial wall of the prepared tooth was 3.1 ± 0.8 mm mesially and 3.0 ± 0.9 distally; thus, the height of the axial wall could be inadequate for the optimal retention of the restoration (Table 1). Furthermore, the optical source of an IOS can barely reach the coordinates of narrow and deep points; consequently, scan data are inaccurate [12]. As such, the scanning inaccuracy increased in the present study when the proximal axial wall height of the prepared tooth was ≥3.0 mm. Nonetheless, in clinical practice, considering that molars must have a proximal axial wall height of ≥4 mm for the retention of restorations, the present results suggested that scanning accuracy should be improved with an increased field of view in the IOS.

Previous studies reported the differences in scanning accuracy with different types of IOSs [13,14,15]. These differences may be due to the mechanical configuration of the optical system of the IOS and the technological variations in the software used for aligning the scanned data and reconstructing the dental arch [16]. Similarly, in the present study, the scan accuracy of the two IOSs significantly differed; specifically, the results of i700 scans were more accurate than those of CS3600 scans regardless of the type of working casts (*p* < 0.05; Table 2). The superior performance of the i700 scanner might be attributed to its relatively more advanced system than that of the CS3600 scanner. Despite the significant difference between the two IOSs (*p* < 0.05; Table 2), the scan accuracy was less than the mean of 30 µm across all data from the two IOSs. Considering the cement space required for a fixed dental prosthesis, studies have recommended a scan data accuracy of <100 µm [20]. Thus, in the present study, both IOSs showed an excellent scan accuracy. However, since the working casts included single-tooth preparations of molars and had a small scan range, further studies on preparations for short- or long-span prostheses are necessary.

This study has minor limitations. First, the 10 working casts were randomly obtained from a clinical setting. Further studies should use varied tooth preparations and scan ranges. Many studies have evaluated various types of tooth preparations and short- or long-span conditions other than single-tooth preparations [17,18]. In addition, the present in vitro study could not reflect numerous factors that might influence the conditions in the dynamic oral cavity of a patient. Factors such as saliva [18], ambient light [20], temperature, humidity [21], types of teeth, and types of tooth preparation can affect the accuracy of intraoral scans; thus, these factors should be considered in future studies. The standardization of abutment dimension and size with a customizable distance from the adjacent teeth should also be investigated [22].

## 5. Conclusions

This study demonstrated that the scan accuracy of IOSs can be influenced by not only the distance between the prepared tooth and adjacent teeth, but also the height of the proximal axial wall of the prepared tooth. For an enhanced accuracy of intraoral scans for tooth preparations, the minimum distance between the adjacent tooth and the prepared tooth should be at least 2.0 mm, and the height of the proximal axial wall of the prepared tooth should be <3.0 mm. However, with the excellent scan accuracy for scanning single-tooth preparations (<30 µm) regardless of the IOS type and differences in working casts, these recommendations may not be strictly necessary. Nevertheless, these findings can help clinicians perform more precise intraoral scanning. The findings further suggest that while specific thresholds for inter-tooth distance and proximal axial wall height can enhance scan accuracy, variability in clinical presentations and scanner capabilities may necessitate individualized adjustment to these parameters.

## Figures and Tables

**Table 1:** Distance between the adjacent teeth and the prepared tooth and axial wall height.

Abutment No.	Distance from the Adjacent Teeth		Axial Wall Height	
	Mesial	Distal	Mesial	Distal
	Distance (Mean ± SD, mm)			
1	2.2 ± 0.3	2.3 ± 0.1	4.6 ± 0.1	2.7 ± 0.2
2	1.1 ± 0.3	1.4 ± 0.5	2.3 ± 0.1	2.0 ± 0.1
3	1.4 ± 0.1	2.3 ± 0.4	3.4 ± 0.2	3.9 ± 0.3
4	1.2 ± 0.3	0.9 ± 0.2	2.4 ± 0.1	2.2 ± 0.1
5	2.2 ± 0.2	1.7 ± 0.2	3.3 ± 0.3	3.1 ± 0.4
6	1.9 ± 0.2	1.6 ± 0.1	3.2 ± 0.1	4.5 ± 0.2
7	1.3 ± 0.3	1.8 ± 0.6	3.4 ± 0.4	2.7 ± 0.1
8	1.5 ± 0.3	1.6 ± 0.1	2.1 ± 0.2	2.2 ± 0.1
9	1.7 ± 0.9	1.5 ± 0.2	4.0 ± 0.2	4.5 ± 0.3
10	2.1 ± 0.3	1.8 ± 0.4	2.0 ± 0.1	2.3 ± 0.2
Mean ± SD	1.7 ± 0.4	1.7 ± 0.4	3.1 ± 0.8	3.0 ± 0.9
*p* *	0.532		0.758	

* Significance as determined by the independent *t*-test for comparing the mesial and distal sides (*p* < 0.05). SD, standard deviation.

**Table 2:** Comparison of the accuracy between the two intraoral scanners in the mesial, distal, buccal, and lingual regions.

Abutment No.	IOS	Accuracy (µm)										F	*p* **	Comparison ***
		Overall		Mesial (M)		Distal (D)		Buccal (B)		Lingual (L)				
		Mean	SD	Mean	SD	Mean	SD	Mean	SD	Mean	SD			
1	CS3600	13.3	2.8	13.7	5.1	13.8	2.0	11.8	2.5	13.0	3.4	1.064	0.372	−
	i700	11.6	0.9	13.1	1.1	10.5	0.7	10.5	1.3	14.0	1.5	34.329	<0.001	M = L > D = B
	t	2.176		0.433		5.752		1.771		−0.986				
	*p* *	0.044		0.671		<0.001		0.091		0.336				
2	CS3600	17.8	2.2	41.3	8.4	11.5	1.8	12.3	2.6	18.4	2.9	131.009	<0.001	M > L > D = B
	i700	12.8	1.7	15.3	3.0	12.4	1.2	11.9	2.6	12.7	2.4	6.079	0.001	M > D = B = L
	t	7.031		11.296		−1.477		0.444		5.801				
	*p* *	<0.001		<0.001		0.151		0.661		<0.001				
3	CS3600	16.8	2.3	16.9	2.5	15.4	2.7	16.7	2.7	18.0	2.5	2.532	0.066	−
	i700	13.7	2.3	30.6	2.0	11.1	1.9	9.2	2.8	12.9	3.0	234.426	<0.001	M > L = D > B
	t	3.736		−16.698		4.994		7.408		4.994				
	*p* *	0.001		<0.001		<0.001		<0.001		<0.001				
4	CS3600	17.4	1.0	19.2	1.6	23.2	1.4	13.3	1.5	15.6	1.5	126.183	<0.001	D > M > L > B
	i700	12.7	0.6	14.5	0.9	20.1	1.2	8.4	0.9	9.7	0.5	499.117	<0.001	D > M > L > B
	t	16.137		10.021		6.415		11.104		14.627				
	*p* *	<0.001		<0.001		<0.001		<0.001		<0.001				
5	CS3600	12.6	1.0	13.3	1.6	12.7	0.8	12.5	1.6	11.6	1.1	3.937	0.013	M = D = B > L
	i700	7.7	0.4	9.1	0.8	8.7	0.6	6.4	0.5	5.9	0.4	118.808	<0.001	M = D > L > B
	t	17.779		8.893		15.9		14.083		18.355				
	*p* *	<0.001		<0.001		<0.001		<0.001		<0.001				
6	CS3600	12.4	0.7	13.0	0.8	11.3	0.9	13.7	1.4	11.2	0.7	24.683	<0.001	M = B > D = L
	i700	9.9	0.4	10.9	0.8	10.2	1.3	7.9	0.6	11.0	0.8	38.757	<0.001	M = D = L > B
	t	12.73		7.141		2.407		15.191		0.414				
	*p* *	<0.001		<0.001		0.023		<0.001		0.682				
7	CS3600	15.1	0.6	26.3	1.8	14.4	0.8	13.2	1.0	11.3	0.8	500.803	<0.001	M > D > B > L
	i700	13.1	0.6	29.8	0.9	9.1	0.4	7.2	0.6	8.2	0.8	3605.813	<0.001	M > D > L > B
	t	9.369		−6.854		22.345		19.418		10.605				
	*p* *	<0.001		<0.001		<0.001		<0.001		<0.001				
8	CS3600	14.3	1.4	15.4	2.5	16.7	2.6	13.1	1.7	12.0	1.6	14.716	<0.001	M = D > B = L
	i700	11.7	1.0	23.5	1.2	11.3	1.3	9.9	1.9	8.8	0.7	373.592	<0.001	M > D > B = L
	t	5.712		−11.433		7.105		4.739		7.243				
	*p* *	<0.001		<0.001		<0.001		<0.001		<0.001				
9	CS3600	13.2	1.2	14.2	2.0	16.8	1.9	10.2	1.6	11.9	2.3	31.681	<0.001	D > M > B = L
	i700	8.0	0.2	10.1	0.6	10.7	1.0	5.5	0.5	6.1	0.4	246.021	<0.001	M = D > B = L
	t	16.687		7.545		11.035		10.79		9.73				
	*p* *	<0.001		<0.001		<0.001		<0.001		<0.001				
10	CS3600	11.5	1.1	15.1	2.3	10.9	0.9	9.8	1.3	11.4	0.9	37.337	<0.001	M > D = L > B
	i700	6.6	0.4	10.6	1.3	6.5	1.0	5.2	0.5	6.0	0.4	116.865	<0.001	M > D = L > B
	t	16.126		6.64		12.728		13.024		20.478				
	*p* *	<0.001		<0.001		<0.001		<0.001		<0.001				

* Significance as determined by an independent *t*-test for comparison between the two intraoral scanners (*p* < 0.05). ** Significance as determined by one-way analysis of variance for comparison among the four regions (*p* < 0.05). *** Order among groups was determined by Tukey’s honest significant difference test (*p* < 0.05).

**Table 3:** Comparison of the correlation of intraoral scanning accuracy with the axial wall height and the distance between the adjacent teeth and the prepared tooth.

		Distance			Axial Wall Height		
		0.9–2.3 mm	~1.9 mm	~2.0 mm	2.0–4.6 mm	~2.9 mm	~3.0 mm
Pearson correlation coefficient		−0.521	−0.617	-	−0.126	-	−0.207
Correlation level *		Moderate	Moderate	-	Weak	-	Weak
*p* **		<0.001	<0.001	0.287	0.002	0.167	<0.001
CS3600	Pearson correlation coefficient	−0.530			−0.190		
	Correlation level *	Moderate			Weak		
	*p* **	<0.001			0.001		
i700	Pearson correlation coefficient	−0.504			-		
	Correlation level *	Moderate			-		
	*p* **	<0.001			0.281		

* Correlation between variables described as strong (−0.7 to −0.9 or 0.7 to 0.9), moderate (−0.4 to −0.6 or 0.4 to 0.6), or weak (−0.1 to −0.3 or 0.1 to 0.3). ** Significance as determined by the Pearson correlation analysis (*p* < 0.05).

## Data Availability

The original contributions presented in the study are included in the article, further inquiries can be directed to the corresponding author.
